# An Efficient Root Transformation System for Recalcitrant *Vicia sativa*

**DOI:** 10.3389/fpls.2021.781014

**Published:** 2022-01-07

**Authors:** Vy Nguyen, Iain R. Searle

**Affiliations:** School of Biological Sciences, The University of Adelaide and Shanghai Jiao Tong University Joint International Centre for Agriculture and Health, The University of Adelaide, Adelaide, SA, Australia

**Keywords:** common vetch, *Vicia sativa*, *Agrobacterium rhizogenes*, hairy root, transformation, legume

## Abstract

Common vetch (*Vicia sativa*) is a multi-purpose legume widely used in pasture and crop rotation systems. Vetch seeds have desirable nutritional characteristics and are often used to feed ruminant animals. Although transcriptomes are available for vetch, problems with genetic transformation and plant regeneration hinder functional gene studies in this legume species. Therefore, the aim of this study was to develop a simple, efficient and rapid hairy root transformation system for common vetch to facilitate functional gene analysis. At first, we infected the hypocotyls of 5-day-old *in vitro* or *in vivo*, soil-grown seedlings with *Rhizobium rhizogenes* K599 using a stabbing method and produced transgenic hairy roots after 24 days at 19 and 50% efficiency, respectively. We later improved the hairy root transformation *in vitro* by infecting different explants (seedling, hypocotyl-epicotyl, and shoot) with *R. rhizogenes*. We observed hairy root formation at the highest efficiency in shoot and hypocotyl-epicotyl explants with 100 and 93% efficiency, respectively. In both cases, an average of four hairy roots per explant were obtained, and about 73 and 91% of hairy roots from shoot and hypocotyl-epicotyl, respectively, showed stable expression of a co-transformed marker β-glucuronidase (GUS). In summary, we developed a rapid, highly efficient, hairy root transformation method by using *R. rhizogenes* on vetch explants, which could facilitate functional gene analysis in common vetch.

## Introduction

Common vetch (*Vicia sativa*) is a multi-purpose legume crop that is widely used in pastures (Sattell et al., 1998), intercropping and crop rotation regimes. Vetch is grown mainly in Europe, Asia, North America, and Oceania, with 54% of the production originating in Europe (FAO data, 1994–2017). Vetch seed is rich in protein, up to 32%, and has very low amounts of lipids making it an appealing food source (Mao et al., 2015). Moreover, symbiotic nitrogen fixation from the atmosphere to the plant is beneficial in crop rotation systems by reducing the amount of nitrogen fertilizer required (Hargrove, 1986). Vetch also exhibits considerable drought tolerance (Tenopala et al., 2012) which is highly valued, particularly as farming in arid areas increases due to climate change (Lobell and Gourdji, 2012).

In recognition that vetch has high potential for genetic improvement, several breeding programs have been established in Europe and Australia. These programs mainly use conventional breeding methods, selecting for traits such as yield improvement (Mikić et al., 2019), non-pod shattering (Abd El-Moneim, 1993), soft seed and rust resistance (Grains Research and Development Corporation [GRDC], 2018). Current research focuses on understanding the molecular basis of important agricultural traits, aiming to facilitate molecular plant breeding (Moose and Mumm, 2008). The recent emergence of transcriptome data (Kim et al., 2015; Dong et al., 2017) will accelerate the identification of genes controlling traits such as pod-shattering (Dong et al., 2017), γ-glutamyl-β-cyanoalanine toxin accumulation (Kim et al., 2015), and drought tolerance (De la Rosa et al., 2020). However, it is difficult to demonstrate the function of candidate genes in vetch due to the lack of a robust transformation and plant regeneration system (Ford et al., 2008; Nguyen et al., 2020). Preliminary research by Maddeppungeng (2006) showed that vetch cells are able to be transformed as evident from green fluorescent protein (GFP) expression in callus, but no regeneration of plants from these transformed cells was reported. Maddeppungeng also reported the formation of embryogenic callus and subsequent plant regeneration from epicotyl explants (Maddeppungeng, 2006), but this has not been confirmed by others. Regeneration of transformed cells is often a problem in many plant species, especially legumes (Somers et al., 2003). Although this can sometimes be overcome by using different varieties, tissue types, media components and culture conditions, the methods are very time consuming and laborious and success is by no means guaranteed.

An alternative to regenerating transgenic plants is to produce transformed hairy roots using the bacterium *Rhizobium rhizogenes*. Examples of success include *Glycine max* (Cho et al., 2000; Kereszt et al., 2007; Cai et al., 2015; Fan et al., 2020), *Vicia hirsuta* (Quandt et al., 1993), *Vicia faba* and *Medicago truncatula* (Vieweg et al., 2004). Previously, the production of transgenic hairy roots were described in vetch by Tepfer (1990), but no detailed description of the method was included. Hairy root induction requires the infection of pluripotent cells with *R. rhizogenes*, transfer and incorporation of the transfer DNA (T-DNA) from the root-inducing plasmid (pRi) into the plant genome and expression of *rol* genes, *rolA*, *rolB*, *rolC, rolD*, encoded on the pRi T-DNA. This often leads to the formation of roots with a “hairy” highly branched phenotype and the loss of plagiotropism (Tepfer, 1984; Sarkar et al., 2018; Tong et al., 2018). Including a second vector carrying a T-DNA containing genes of interest in the bacteria can lead to co-transformation into the plant nuclear genome (Tepfer, 1990). Co-transformation of up to 88% was reported in *V. hirsuta* (Quandt et al., 1993) but efficiency ranges from 20 to 80% in other plants (Kereszt et al., 2007). Considering the possibility of using *R. rhizogenes* transformation, we aimed to develop a reliable, high-efficiency and time-saving protocol for vetch hairy root transformation to facilitate functional gene analysis in vetch.

In this study, we first adapted a method from Kereszt et al. (2007) for hairy root induction by stabbing *R. rhizogenes* strain K599 into the hypocotyl region of 5-day old seedlings. Twenty-four days after inoculation of *in vitro* or soil-grown seedlings, about 19 and 50%, respectively, of the infected seedlings produced transformed hairy roots. Due to the low efficiency of this method under *in vitro* conditions, we attempted to improve the transformation efficiency by using different tissue explants. We observed hairy root formation at the highest rate in shoot and hypocotyl-epicotyl tissues with 100 and 93% efficiency, respectively. In both cases, an average of four hairy roots per explant was obtained and about 73 and 91% of the hairy roots from shoot and hypocotyl-epicotyl tissues, respectively, showed stable expression of a co-transformed marker gene encoding β-glucuronidase (GUS).

In summary, we successfully adapted hairy root induction using *R. rhizogenes* strain K599 to vetch and generated hairy roots *in vitro* with high efficiency. An *in vitro* hairy root induction experiment for vetch took less than a month, and transgenic hairy roots could be obtained as early as 12 days after the infection. Moreover, our simplified inoculation medium, RGM_NoSuc, helped eliminate the use of an expensive antibiotic while successfully controlling the *R. rhizogenes* overgrowth during hairy root establishment. This medium was able to maintain the hairy root growth for at least 4 weeks after infection. Finally, we show that hairy roots may be regenerated directly from shoot tissue, and these roots are sufficient to support shoot growth. Therefore, this will be a valuable tool for plant propagation in species with poor root establishment *in vitro*, such as vetch in our case.

## Materials and Methods

### Plasmids Containing Genes for Overexpression of β-Glucuronidase and Green Fluorescent Protein

Plasmids for overexpression of GUS (pSB161, Addgene #123197) and GFP (pSB115, Addgene #123190) were obtained from Addgene and transformed into *R. rhizogenes* strain K599 *via* electroporation (Mortensen et al., 2019).

### Preparing *R. rhizogenes* Cultures

*Rhizobium rhizogenes* cells were stored at −80°C. Before vetch transfection, the bacteria were streaked onto an LB plate or LB plate supplemented with 50 mg/L kanamycin to select for the presence of pSB161 or pSB115 plasmids (Mortensen et al., 2019). A single colony was then inoculated into a 3 mL LB liquid culture and shaken at 180 rpm at 30°C in the dark. The next day, 1 mL of the culture was mixed with 200 μl of 80% glycerol, and 200 μl of the mixture was spread onto an LB plate supplemented with kanamycin. The bacteria were cultured overnight in the dark at 30°C.

### Seed Sterilization for *in vitro* Culture

Vetch (*V. sativa* cultivar Studenica) seeds were surface sterilized with 70% EtOH for 1 min, followed by 5% NaOCl for 20 min with vigorous agitation, and then rinsed with sterilized reverse osmosis water for 1 min and repeated four more times. The seeds were then sown on RGM_NoSuc medium (Table 1) and grown under fluorescent lights (1000 μmol m^–2^ s^–1^) with a 16 h photoperiod at 25°C.

**TABLE 1:** Media formulation.

Component	Sigma-Aldrich Cat. Number	RGM_NoSuc*	RGM_3xSuc
MS (Murashige and Skoog Basal) Media	M5519	2.2 g/L	2.2 g/L
1000x MS vitamins	M3900	1 mL/L	1 mL/L
MES hydrate	M2933	0.5 g/L	0.5 g/L
Sucrose	S0389	0 g/L	30 g/L
Agar	A1296	10 g/L	
Gelzan	G1910		3 g/L
Adjust pH with 1 M KOH		5.7	5.7

*Inoculation media.

### Vetch Transformation on Soil Using a Seedling Stabbing Method

Vetch (*V. sativa* cultivar Studenica) seeds were sown 2 cm apart in a tray (30 cm × 50 cm × 15 cm) containing a mixture of sterile sand and cocopeat (1:1 in volume) and covered with a 1 cm layer of vermiculite. The seedlings were then grown under halogen lights (1000 μmol m^–2^ s^–1^) with a 16 h photoperiod at 25°C (David et al., 2017). Five days after sowing, a small paste of *R. rhizogenes* was picked from the plate using a 27G 1/2 (0.4 mm × 13 mm) needle and injected into the hypocotyl region of seedlings. The bacteria were pushed into the middle of the hypocotyls by inserting the needle three times. Next, the infected regions were covered with vermiculite and watered with mist from a spray bottle. A transparent cover was placed over the tray to maintain high humidity. Water was sprayed regularly to keep the vermiculite layer moist. Nine days after infection (DAI), adventitious roots emerged from the wounded sites and were trimmed off to promote the development of hairy transformed roots. Twenty-four DAI, hairy roots were observed and harvested for further analysis.

### Vetch Transformation *in vitro* Using a Seedling Stabbing Method

*Rhizobium rhizogenes* was introduced into the hypocotyl region of a 5-day old *in vitro* seedling using a similar stabbing technique to the on-soil method described above. After infection, the seedlings were cultured on filter paper (optional) on a petri dish with RGM_NoSuc media (Table 1) under fluorescent lights (1000 μmol m^–2^ s^–1^) with a 16 h photoperiod at 25°C until hairy roots were observed.

### Vetch Transformation *in vitro* Using Hypocotyl-Epicotyl Explants

The primary shoot and roots of a 5-day-old *in vitro* seedling were trimmed off, and the middle region containing the hypocotyl-epicotyl attached to the seed was used for infection with *R. rhizogenes*. A surgical blade coated with *R. rhizogenes* was used to cut longitudinally into the middle region of the hypocotyl-epicotyl. The explant was then laid on RGM_NoSuc inoculation medium and cultured under fluorescent lights (1000 μmol m^–2^ s^–1^) with a 16 h photoperiod at 25°C until hairy root emerged at approximately 12 DAI.

### Vetch Transformation *in vitro* Using Shoot Explants

Stems with a lateral or apical shoot were isolated from a 1-month-old *in vitro* plant by using a surgical blade coated with *R. rhizogenes.* The infected explants were then laid down onto RGM_NoSuc inoculation medium with the infected sites touching the medium. The explants were then cultured under fluorescent lights (1000 μmol m^–2^ s^–1^) with a 16 h photoperiod at 25°C until hairy root emerged at approximately 12 DAI.

### Maintaining Hairy Root Culture *in vitro*

Hairy roots were isolated from original explants at 24 DAI and were sub-cultured on RGM_3xSuc medium for 4 weeks (Table 1) with 25 mg/L meropenem added (Sigma-Aldrich) to eliminate *R. rhizogenes*. The hairy roots were then sub-cultured in new RGM_3xSuc every 4 weeks and cultured in the dark at 25°C.

### Growth of Transgenic Hairy Root Composite Plants on Soil

Twenty-four DAI, an *in vitro* composite plant containing a wild-type shoot and transgenic hairy roots was transferred onto the soil (Seed raising mix, Debco). A plastic cover was placed on top to maintain high humidity for about 2 weeks. After that, the propagation cover was removed, and watering was adjusted according to the plant’s need. Plants were grown under halogen lights (1000 μmol m^–2^ s^–1^) with a 16 h photoperiod at 25°C.

### PCR Confirmation of Transgenic Hairy Roots

The hairy root phenotype is caused by the combined expression of *rol* genes, *rolA-rolD*, from the K599 T-DNA of pRi2659 after incorporation into the nuclear genome. To detect the pRi T-DNA in the vetch genome, *rolB* specific PCR primers, *rolB*_F- and *rolB*_R, were used to PCR amplified a 381 bp product from transgenic hairy roots (Table 2).

**TABLE 2:** Oligonucleotide PCR primers used in this study.

Primer	Sequence (5′ – 3′)	Target
rolB_F	ccctcttcacgtttctggttgg	rolB*
rolB_R	gtttggattagaggccgtgctg	

*PCR product is 381 bp.

### A Histochemical β-Glucuronidase Expression Assay for Transgenic Hairy Roots

Hairy roots were incubated with substrate solution, 2 mM XGluc (cat. G1281C1, Gold Biotechnology), 0.5 mM K_3_Fe_6_, 0.5 mM K_4_Fe_6_, 50 mM NaPO_4_ pH 7.2 and 0.1% (v/v) Triton X-100 at 37°C overnight as described by Jefferson et al. (1987). The roots were then washed with 100% EtOH three times and incubated in EtOH for at least 1 h prior to observation under a stereomicroscope (Olympus SZ2_ILST).

### Green Fluorescent Protein Detection in Transgenic Hairy Roots

Green fluorescent protein expressing transgenic hairy roots were observed by using a hand-held, filtered blue light (Dark Reader Lamp – Clare Chemical Research). GFP expressing tissue appeared green, and tissue containing chlorophyll appeared red due auto-fluorescence.

## Results

### Hairy Root Induction on Soil Using a Seedling Stabbing Method

**FIGURE 1:**
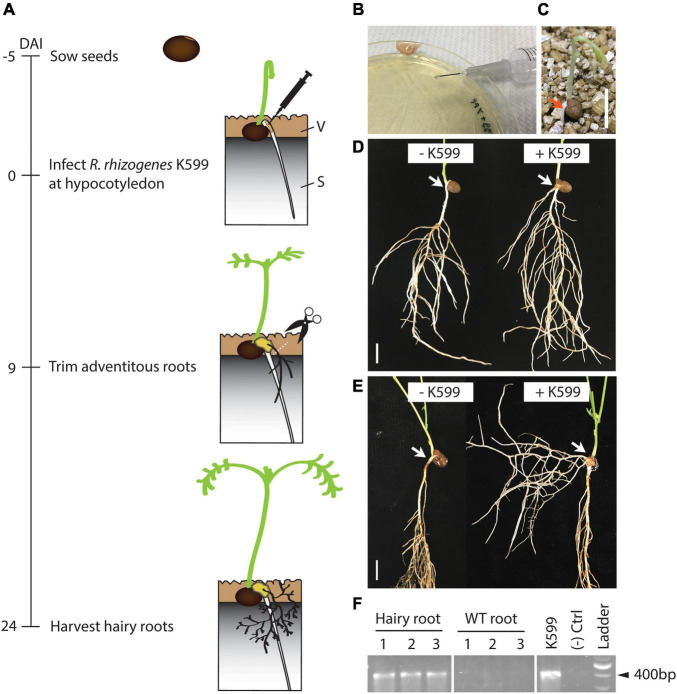
Hairy root induction from vetch hypocotyl on soil by *R. rhizogenes* K599 using the stabbing method. **(A)** Timeline for hairy root induction on soil by *R. rhizogenes*. Vetch seeds were sown on top of a mixture of sand and cocopeat (1:1), and 1 cm of vermiculite was placed on top to cover the seeds (5 days before infection). On day 5, seedling hypocotyl regions were infected with *R. rhizogenes* (shown as day 0). Nine days after infection (DAI), adventitious roots were observed at the infected region and were trimmed off to promote the growth of hairy roots. Twenty-four DAI, hairy roots were observed. **(B)** *R. rhizogenes* were grown on an LB plate and scraped into a paste which was pushed into the hypocotyl region of plants by stabbing with a 27G 1/2 needle. **(C)** A 5-day old seedling with the red arrow showing the position to be inoculated using the stabbing method. **(D)** A 9 DAI control seedling that was stabbed with a needle containing no bacteria (–K599) and a seedling infected with K599 (+K599). White arrow indicates inoculation site. No callus developed at the wounded site on the control plant. +K599 plant showed the formation of callus and adventitious roots at the wounded site. However, the adventitious roots had a wild type phenotype and were trimmed to promote hairy root development. **(E)** At 24 DAI, –K599 plant showed no callus or roots at the wounded sites. In. contrast +K599 plant showed the formation of hairy roots, which were highly branched and had lost plagiotropism. **(F)** The *rolB* gene was PCR amplified from DNA isolated from hairy roots but not from non-hairy roots from the same plants (three biological replicates, *n* = 3, are shown). DAI, day after the infection; V, vermiculite; S, sand and cocopeat (1:1, volume:volume). Scale bar = 1 cm.

To determine whether *R. rhizogenes* K599 strain can trigger vetch hairy root formation, we adapted a previously described soybean hairy root production method (Kereszt et al., 2007; Fan et al., 2020). In our adaptation, 5-day-old soil-grown vetch seedlings were infected with *R. rhizogenes* by pushing the bacterial inoculum into the hypocotyl region using a small gauge needle. High humidity was maintained at the wound site by keeping a surrounding vermiculite layer moist all times (Figure 1A). Nine days after inoculation (DAI), callus and adventitious roots were observed at the wound site (Figures 1B–D) and they did not have the required hairy root phenotype (for example, highly branching, or loss of plagiotropism). In the negative control plant, wounds were introduced but there was no callus formation without the bacteria (Figure 1D). However, a few control plants did show adventitious roots protruding from the wounded sites (not shown). We considered that the first adventitious roots 9 DAI might be just the result of wounding, so these roots were trimmed to promote the growth of transgenic hairy roots. Twenty-four DAI, roots protruding from the callus at the infected sites showed a typical hairy root phenotype: highly branched and showing the loss of plagiotropism (Figures 1A,E and Supplementary Figure 1). These roots were absent in the negative control plant (Figure 1E). PCR detection was performed to confirm the presence of the *rol* genes in the hairy roots. The PCR results showed the presence of *rolB* gene in the hairy roots tested, but there were no PCR products in the non-hairy roots from the same plant (Figure 1F).

### Hairy Root Induction *in vitro* Using a Seedling Stabbing Method

**FIGURE 2:**
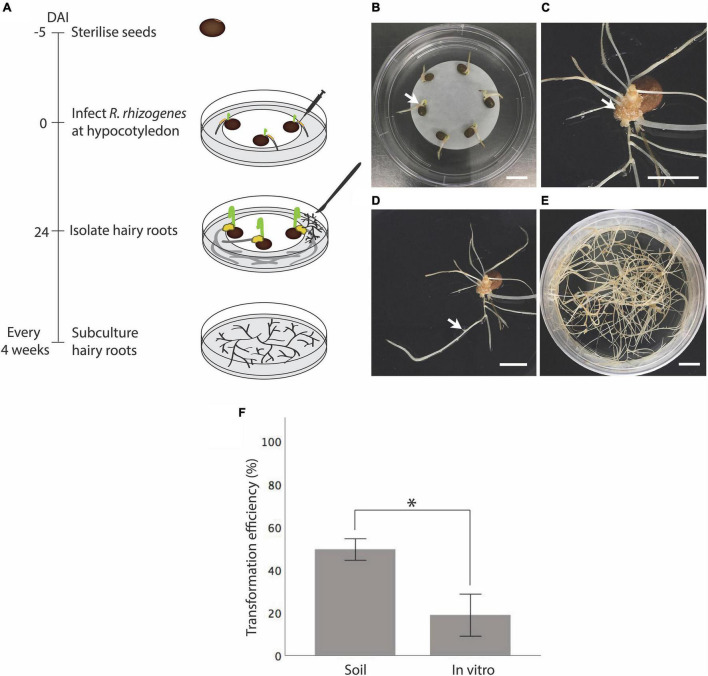
*In vitro* hairy root induction from vetch hypocotyl by *R. rhizogenes* K599 using the stabbing method. **(A)** Timeline for *in vitro* hairy root induction by *R. rhizogenes*. On day 1, sterilized seeds were sown on a plate containing inoculation (RGM_NoSuc) medium and placed at 25°C until germination. On day 5, the seedling hypocotyl region was inoculated with *R. rhizogenes* K599 using the stabbing method and then cultured on inoculation medium with a filter paper (optional) under fluorescent light at 25°C. Twenty-four days after the infection (DAI), hairy roots were isolated and subcultured onto RGM_3xSuc medium with 25 mg/L meropenem. Hairy roots were subcultured every 4 weeks onto fresh medium. **(B)** Inoculation of seedling hypocotyl regions (white arrow) with *R. rhizogenes.* **(C)** Twenty-four DAI, callus developed from the inoculated region (white arrow). **(D)** A hairy root protruded from the callus (white arrow). Early lateral root formation in the hairy root created a highly branched structure, which could help to distinguish hairy roots from wild type roots. **(E)** Hairy roots 45 DAI were highly branched. **(F)** On soil and *in vitro* hairy root induction efficiency when using seedling hypocotyl stabbing method (*n* = 20 for each medium). *Statistically significant at 0.05, *t*-test (SPSS statistics V.20). Error bar = 95% CI. DAI, days after the infection. DNA ladder is 100 bp ladder (Thermo FISHER SCIENTIFIC). Scale bar = 1 cm.

To produce vetch hairy roots under sterile conditions, we further modified the on soil hairy root induction method. First, sterilized seeds were germinated on RGM_NoSuc medium and grown at 25°C for 5 days. Then *R. rhizogenes* was infected into the hypocotyl region similarly to the on-soil stabbing method, but in a sterile laminar flow hood (Figure 2A). To avoid bacterial overgrowth, RGM_NoSuc inoculation medium without sucrose was used, and a filter paper was initially placed underneath the seedling so that its roots were still touching the medium (Figures 2A,B). However, we later found that the filter paper could be eliminated as medium lacking sucrose was sufficient to suppress the bacterial overgrowth. The seedlings were then cultured under lights on the same medium. Callus formation was observed at the wounded sites (Figure 2C), and at 24 DAI, hairy roots were observed (Figure 2D). Compared with wild type roots, hairy roots showed very early lateral root formation and vigorous growth. As a result, hairy roots were much more branched when compared with wild type roots (Figure 2E). Moreover, due to the loss of geotropism, hairy roots not only penetrated the medium but also grew upward (not shown). A comparison between methods shows that roughly 50% of seedlings were confirmed to successfully produce transgenic hairy roots after the infection with K599 on soil, while this had dropped to only 19% when performed *in vitro* (Figure 2F and Supplementary Table 1).

### Hairy Root Induction *in vitro* Using Hypocotyl-Epicotyl and Shoot Explants

**FIGURE 3:**
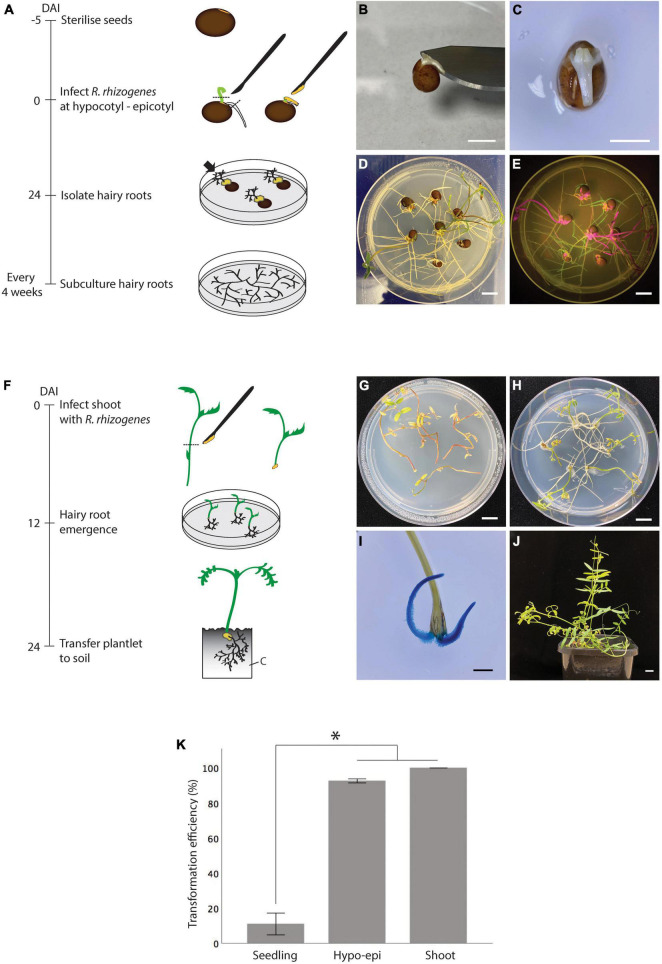
*In vitro* hairy root induction from vetch hypocotyl-epicotyls and shoots by *R. rhizogenes* K599 using the cutting method. **(A)** Timeline for *in vitro* hairy root induction from hypocotyl-epicotyl tissues by *R. rhizogenes*. On day 1, sterilized seeds were sown on a plate containing RGM_NoSuc medium and germinated at 25°C. On day 5, primary shoots and roots of the seedlings were trimmed off, and hypocotyl-epicotyl regions were infected with *R. rhizogenes* by making a longitudinal cut with a surgical knife containing the bacterial paste. Infected explants were cultured on RGM_NoSuc medium under fluorescent light at 25°C. Twenty-four days after the infection (DAI), hairy roots were isolated and subcultured. The black arrow indicates hairy root growth from the inoculation site. **(B)** After trimming off a primary root and shoot, the remaining hypocotyl-epicotyl attached to the seed was infected by cutting longitudinally into the middle part of it with a surgical blade carrying the bacteria. **(C)** View from above of a hypocotyl-epicotyl explant after the infection. **(D)** Hairy root formation from the infected hypocotyl-epicotyl explants at 24 DAI. **(E)** GFP-expressing transgenic hairy roots (green color) were detected using a hand-held blue light (Dark Reader Lamp – Clare Chemical Research), red color is the auto-florescence from tissue containing chlorophyll. **(F)** Timeline for *in vitro* hairy root induction from shoot by *R. rhizogenes.* On day 1, shoots and internodes containing a shoot were isolated and infected with bacteria at the basal pole. Infected shoots were laid onto RGM_NoSuc medium so that the infected regions were touching the medium. Twelve days after the infection (DAI), the hairy roots on the explants were observed. Twenty-four DAI, a shoot with newly regenerated hairy roots can be transferred onto the soil. **(G)** Non-infected shoots became withered at 24 DAI as no root development to support the shoot growth. **(H)** Infected shoots with hairy root formation at 24 DAI. **(I)** Confirmation of hairy root regenerated from the shoot at 12 DAI using GUS marker. **(J)** Hairy roots were able to support shoot growth, and composite plantlets survived under non-sterile condition. **(K)** *In vitro* hairy root induction efficiency of hypocotyl-epicotyl (Hypo-epi) and shoot cutting methods compared to hypocotyl stabbing method (*n* = 25). *Statistically significant at 0.05, *t*-test (SPSS statistics V.20). Error bar = 95% CI. DAI, day after the infection; C, cutting mix. Scale bar **(B,C)** = 0.5 cm; **(D,E,G,H,J)** = 1 cm; **(I)** = 2 mm.

Although hairy root induction *in vitro* by stabbing *R. rhizogenes* into the hypocotyl region of a seedling was successful, it was inefficient, only about 19 ± 10%, and replicates varied widely (Figure 2F and Supplementary Table 1). Therefore, we extended the tested explants to hypocotyl-epicotyls (Figures 3A–E) and shoots, aiming to improve the hairy root induction efficiency *in vitro* (Figures 3F–J). To facilitate the identification of explants that developed transgenic hairy roots, we further prepared K599 carrying an overexpressing of GFP (pSB115) or GUS (pSB161) plasmid. Unlike the stabbing method where bacteria were introduced into a small area, infected hypocotyl-epicotyl and shoot explants required dissecting the hypocotyl-epicotyls and shoots using a K599 coated surgical blade which resulted in a larger infected area (Figures 3B,C). For comparison, the hypocotyl stabbing method was performed for seedlings again in this experiment. As predicted, the hairy root induction efficiency from seedlings was low, only 11% (Figure 3K and Supplementary Table 2). On the other hand, 93% hypocotyl-epicotyl and 100% shoot developed hairy roots at 24 DAI (Figure 3K and Supplementary Table 2).

### Co-transformation Efficiency in Hairy Root

**FIGURE 4:**
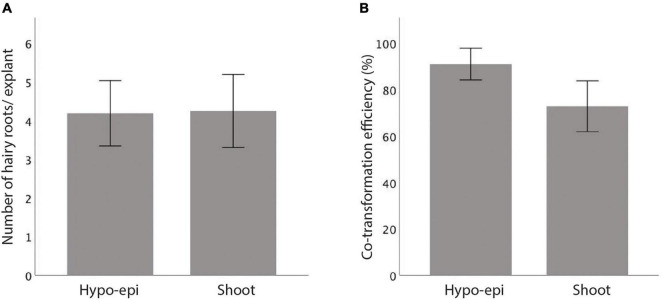
*In vitro* hairy root induction comparison for vetch hypocotyl-epicotyl (Hypo-epi) and shoot after infected with *R. rhizogenes* K599. **(A)** An average number of hairy roots per explant formed 24 days after the infection. **(B)** Percentage of GUS expressing hairy root in total hairy root per explant (co-transformation efficiency, *n* = 40). Error bar: 95% CI. **(A,B)** No statistically significant at 0.05, *t*-test (SPSS statistics V.20).

When *R. rhizogenes* K599 strain infects a plant cell, the T-DNA containing the *rol* genes is incorporated into the plant genome (Sarkar et al., 2018; Tong et al., 2018). By introducing a second plasmid with a T-DNA region carrying a gene of interest into K599, the same co-transfer process occurs. However, it has been observed that not all the T-DNAs in the bacteria are transferred concomitantly into the plant genome (Fan et al., 2020). Therefore, to estimate the co-transformation efficiency of our K599 strain in vetch, a second plasmid with T-DNA carrying an overexpressed GUS gene was first electroporated into K599 and the bacteria were used to infect vetch hypocotyl-epicotyls and shoots. The explants with hairy roots obtained at 24 DAI were stained with GUS for co-transformation examination. Typically, an average four hairy roots/explant were observed for hypocotyl-epicotyl and shoot transformation (Figure 4A and Supplementary Table 3). Furthermore, an average of about 91% and 73% of hairy roots from hypocotyl-epicotyls and shoots, respectively, showed GUS activity (Figure 4B and Supplementary Table 4).

### The Deterioration of Vetch Hairy Root Growth Under Prolonged Culture *in vitro*

**FIGURE 5:**
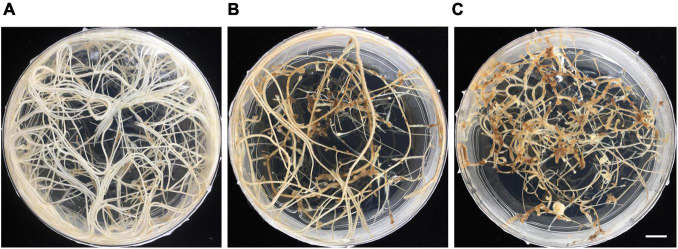
Deterioration of hairy root growth under prolonged culture *in vitro*. **(A)** Two-month-old hairy roots. **(B)** Five-month-old hairy roots. **(C)** Six-month-old hairy roots. Scale bar = 1 cm.

Hairy roots were maintained by sub-culturing onto a new RGM_3xSuc medium every 4 weeks. For each subculture, 5 cm segments of the hairy root, which contained about 5–10 lateral roots were transferred onto new RGM_3xSuc medium. After a 4-week-growth period, we observed extensive root growth that covered the surface of the media in the 9 cm petri dish, as shown in Figure 5A. Vigorous hairy root growth was maintained for about 4 months. After that, a significant reduction in root growth was observed: the roots turned brown, and newly established lateral roots were thin and slow-growing with a swollen root tip (Figures 5B,C).

## Discussion

### Adaptation of an On-Soil Seedling Stabbing Method to *in vitro* Conditions

Previously, the stabbing method was used successfully for soybean, in a non-sterile conditions (Kereszt et al., 2007; Fan et al., 2020). Therefore, we adapted this method aiming to study gene expression in vetch. Our study indicated that the *R. rhizogenes* K599 strain was able to infect vetch and subsequently triggered the infected cell to form hairy roots at 50% efficiency (Figure 2 and Supplementary Table 1). Later, we preferred transgenic tissue to be obtained *in vitro* as with this condition, non-experimental bacterial and fungal contamination and contamination with other organic materials, could be eliminated. The cleanliness of the tissue is vital in, for example, nucleotide sequencing experiments or during the detection of a gene of interest using a sensitive technique such as PCR. However, adapting the stabbing method to *in vitro* conditions showed only 19% efficiency in vetch, and this observation was consistently low for eight independent transformation experiments (Figure 2 and Supplementary Table 1). The possible reason for the low efficiency of this method when performed *in vitro* could be the difference in the poor physical growth of the seedlings between on soil and *in vitro* conditions.

### Removal of Primary Root Could Enhance Hairy Root Formation

One of the possible reasons for the low hairy root induction efficiency in the stabbing method could be the use of a whole seedling. After stabbing, the primary root continued growth, which could inhibit lateral root formation, including hairy roots. Presumably, trimming of the main root could overcome the dominant effect of the primary root and promote the growth of lateral roots and hairy roots from the callus (Fan et al., 2020). Indeed, our epicotyl-hypocotyl explants had their primary shoots and roots removed, and this could promote the formation of hairy roots to replace the loss of primary root (Supplementary Figure 2). Although the primary shoot was removed from epicotyl-hypocotyl explants in our experiment, a vetch seedling has two lateral shoots nested at the junction between the cotyledon and the epicotyl, and these shoots continued their growth during hairy root formation (Supplementary Figure 3). Therefore, removal of the primary root might be more important in promoting hairy root formation.

### High Efficiency Hairy Root Regeneration From the Shoot as a Tool for Vetch Propagation

Root regeneration from the shoots of vetch species by auxin application has been reported, such as in hairy vetch (*Vicia villosa*) (Wiering et al., 2020) and Hungarian vetch (*Vicia pannonica*) (Sahin-Demirbag et al., 2008). However, there is no report regarding root regeneration for common vetch (*V. sativa*). Our attempts to regenerate roots from common vetch shoots using auxin application have failed to date. However, when we infected common vetch shoots with *R. rhizogenes* K599 we observed 100% of the infected shoots regenerating roots (Figures 3H,K). We speculated that *R. rhizogenes* infection interferes with auxin/cytokinin production in vetch tissues, promoting root formation as a result (Bettini et al., 2010; Sarkar et al., 2018).

Previously, hairy roots supporting shoot growth has been reported (Kereszt et al., 2007). Therefore, we transferred the composite vetch from *in vitro* to soil conditions for further observation. So far, the hairy roots have been able to support vetch wild type shoots with no adverse effects on growth (Figure 3J). This straightforward and reliable way for root regeneration could be helpful in vetch propagation and could be applied for other recalcitrant species.

### Co-transformation Efficiency of K599 in Vetch

*Rhizobium rhizogenes* K599 has successfully induced hairy roots in many species, including vetch. However, there is always a question about the probability of the bacteria to co-transform the second binary T-DNA as well as the pRi2659 T-DNA region (Mankin et al., 2007; Lee and Gelvin, 2008; Tong et al., 2018). To test the co-transformation efficiency of *R. rhizogenes* K599 transgenic roots, we generated hairy roots from shoot explants and co-transformed a GUS visual reporter. Using the GUS reporter gene, we confirmed that not all the hairy roots developed from infected shoots expressed GUS activity. The co-transformation efficiency was estimated about 72.9 ± 19.8% for the shoot (Figure 4B and Supplementary Table 4). The co-transformation efficiency might need to be accessed case by case such as the bacterial strain and the plant variety used for transformation as well as the *in vitro* culture conditions. Also, the co-transformation efficiency could be under-estimated as it is known that a high copy number of a T-DNA plasmid may cause multi-insertion into the plant genome, thereby triggering gene silencing (Lee and Gelvin, 2008). Another strategy that could be used to achieve maximum transformation efficiency of a gene of interest and reduce the risk of gene silencing is to use a co-integration/exchange system by inserting the gene of interest into a T-DNA region of pRi2659 in *R. rhizogenes*, such that only this T-DNA would be transferred into the plant (Lee and Gelvin, 2008). It depends on researchers to choose to use T-DNA binary vector system or co-integration/exchange system regarding their workload.

### Controlling Bacteria Overgrown After the Transfection

One of the problems when using bacteria for transformation is to eliminate the bacteria after transfection is completed. Often not long after the infection (usually 3–5 days), explants will be moved onto a new medium supplemented with an antibiotic supplement to suppress the growth of the bacteria, and subsequent subcultures are necessary to eliminate the bacteria (Böttinger et al., 2001; Olhoft et al., 2007). This process is laborious. In this study, we found that eliminating sucrose from the inoculation medium (RGM_NoSuc medium) effectively suppresses the growth of bacteria without inhibiting transformation and hairy root formation. From the first day of the infection to 4 weeks later, hairy roots formed and increased their mass on RGM_NoSuc medium under 16 h light period conditions. The newly formed hairy roots appeared to be free from bacteria, and if carefully removed and subcultured without touching the visible bacteria at the infected sites, these roots remained sterile. In our protocol, we first subcultured hairy roots on RGM_3xSuc with meropenem (25 mg/L) for 4 weeks; after that, another subculture on RGM_3xSuc without meropenem showed no sign of bacterial growth suggesting that the roots were free from bacteria. Although eliminating sucrose from the medium helped control the bacterial overgrowth, the hairy roots developed on this medium were thin and slow growing. Adding sucrose to the medium helped to enhance the growth of the roots. Therefore, after successfully establishing hairy roots, the roots were subcultured onto medium with added sucrose (RGM_3xSuc).

### Agar Versus Gelzan in Hairy Root Culture

Gelzan or gellan gum, is a gelling agent that can replace agar in growth medium (Grasdalen and Smidsrød, 1987; Paulraj and Yeung, 2012). Compared with agar, gelzan medium is more transparent, which was helpful for observing roots. Moreover, gelzan medium was softer than agar medium, making it easier to remove the roots from the medium without damage. However, during the bacterial inoculation step, medium with gelzan (3 g/L) appeared wet, possibly increasing the risk of necrosis at the infected site. In our experience, using agar at a concentration 10 g/L was best suited for the inoculation step.

### Deterioration of Hairy Root Growth Under *in vitro* Conditions

It was not surprising to observe in vetch that hairy root growth decreased after a few months of *in vitro* culture as vetch is an annual plant. The underlying molecular causes of the decreased growth are unclear but may be due to epigenetic changes that are inherited over prolonged culture periods. This suggests that the hairy root material from vetch and other annual plants should be used within 3 months after infection to ensure the highest quality root material.

## Conclusion

Establishment of transgenic hairy roots using *R. rhizogenes* strain K599 was successful for Common Vetch. New explant material (epicotyl-hypocotyl and shoot) and inoculation medium (RGM_NoSuc) improved hairy root induction efficiency and reduced the time and labor needed for tissue culture. Our simple method could also produce contaminant-free transgenic hairy roots for downstream study. Additionally, root regeneration for recalcitrant vetch could be made more efficient by applying *R. rhizogenes* to shoot tissue. This simple way of root induction from shoots could be helpful in plant propagation.

## Data Availability Statement

The original contributions presented in the study are included in the article/Supplementary Material, further inquiries can be directed to the corresponding author.

## Author Contributions

IS conceived the project, interpreted the results, and edited the manuscript. VN conducted the experiments, interpreted the results, and drafted the manuscript. Both authors contributed to the article and approved the submitted version.

## Conflict of Interest

The authors declare that the research was conducted in the absence of any commercial or financial relationships that could be construed as a potential conflict of interest.

## Publisher’s Note

All claims expressed in this article are solely those of the authors and do not necessarily represent those of their affiliated organizations, or those of the publisher, the editors and the reviewers. Any product that may be evaluated in this article, or claim that may be made by its manufacturer, is not guaranteed or endorsed by the publisher.

## Supplementary Material

Supplementary Figure 1 Hairy root induction on soil 24 days after infection with *R. rhizogenes* K599. **(A)** A highly branching hairy root (white arrow). **(B)** Hairy root emerged to the soil surface due to loss of plagiotropism (white arrow). Scale bar = 1 cm.

Supplementary Figure 2 GFP hairy root induction from **(A)** hypocotyl-epicotyl and **(B)** stabbed seedling. **(A)** Primary root was removed at day 0 of the infection with *R. rhizogenes*. **(B)** Primary root was kept during the hairy root induction period. GFP expressing transgenic hairy roots (green color) were detected using a hand-held blue light (Dark Reader Lamp – Clare Chemical Research), red color was the auto-florescence from tissue containing chlorophyll. White arrow indicates the wild type primary root. Scale bar = 1 cm.

Supplementary Figure 3 Lateral shoots of vetch seedling. **(A)** Vetch seedling 5 days after germination *in vitro* on RGM_NoSuc media. **(B)** A close-up of a lateral shoot. Black arrows indicate the lateral shoot position at the junction of cotyledon and epicotyledon. Scale bar **(A)** = 1 mm; **(B)** = 0.5 mm.
