# Supplementary figures and images for: An Efficient Root Transformation System for Recalcitrant Vicia sativa
Source: Front Plant Sci. 2022 Jan 7;12:781014. doi: 10.3389/fpls.2021.781014 (PMC8777216; doi:10.3389/fpls.2021.781014)

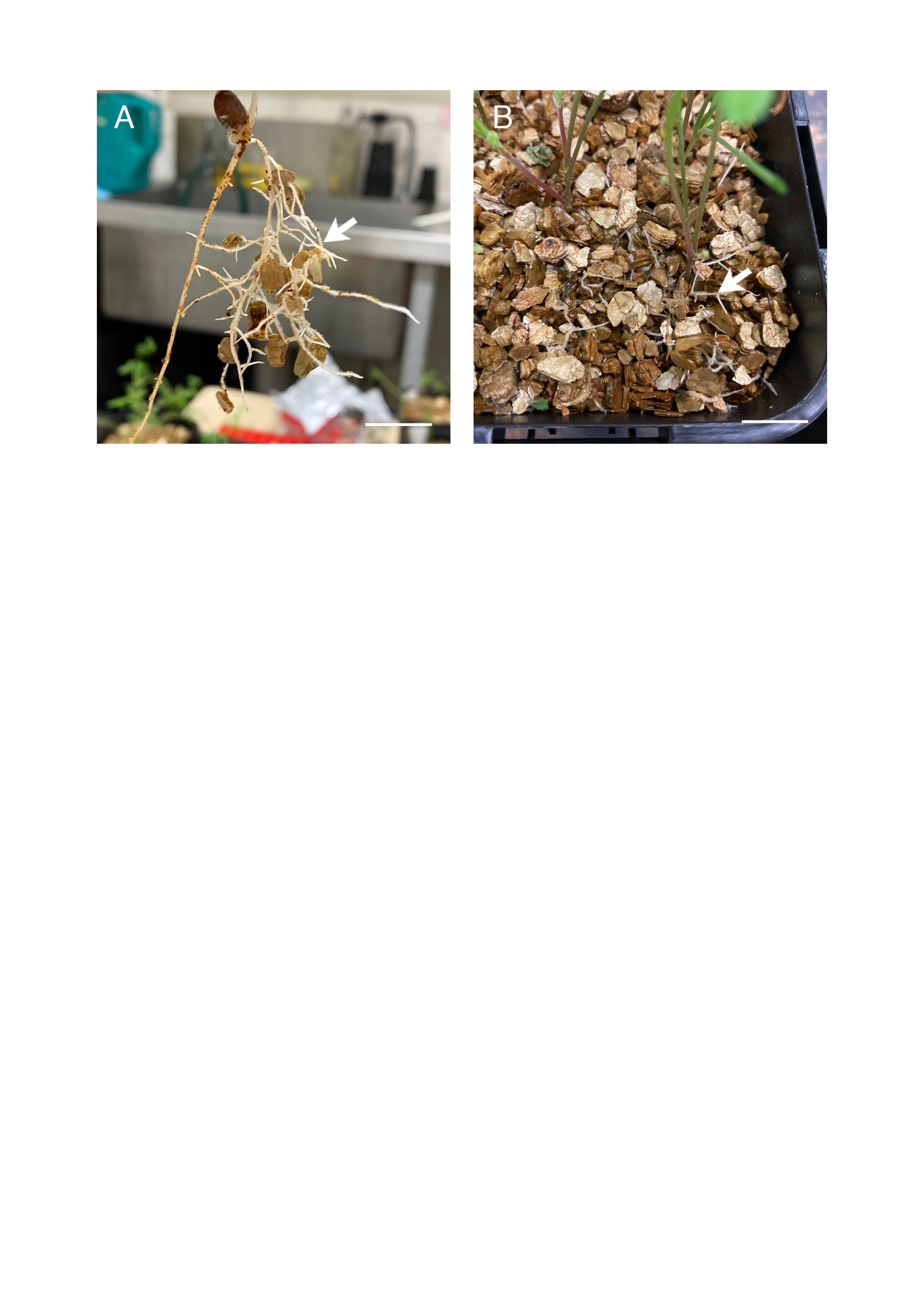

Supplement: Supplementary Figure 1 — Hairy root induction on soil 24 days after infection with R. rhizogenes K599. (A) A highly branching hairy root (white arrow). (B) Hairy root emerged to the soil surface due to loss of plagiotropism (white arrow). Scale bar = 1 cm. [file Image_1.JPEG]

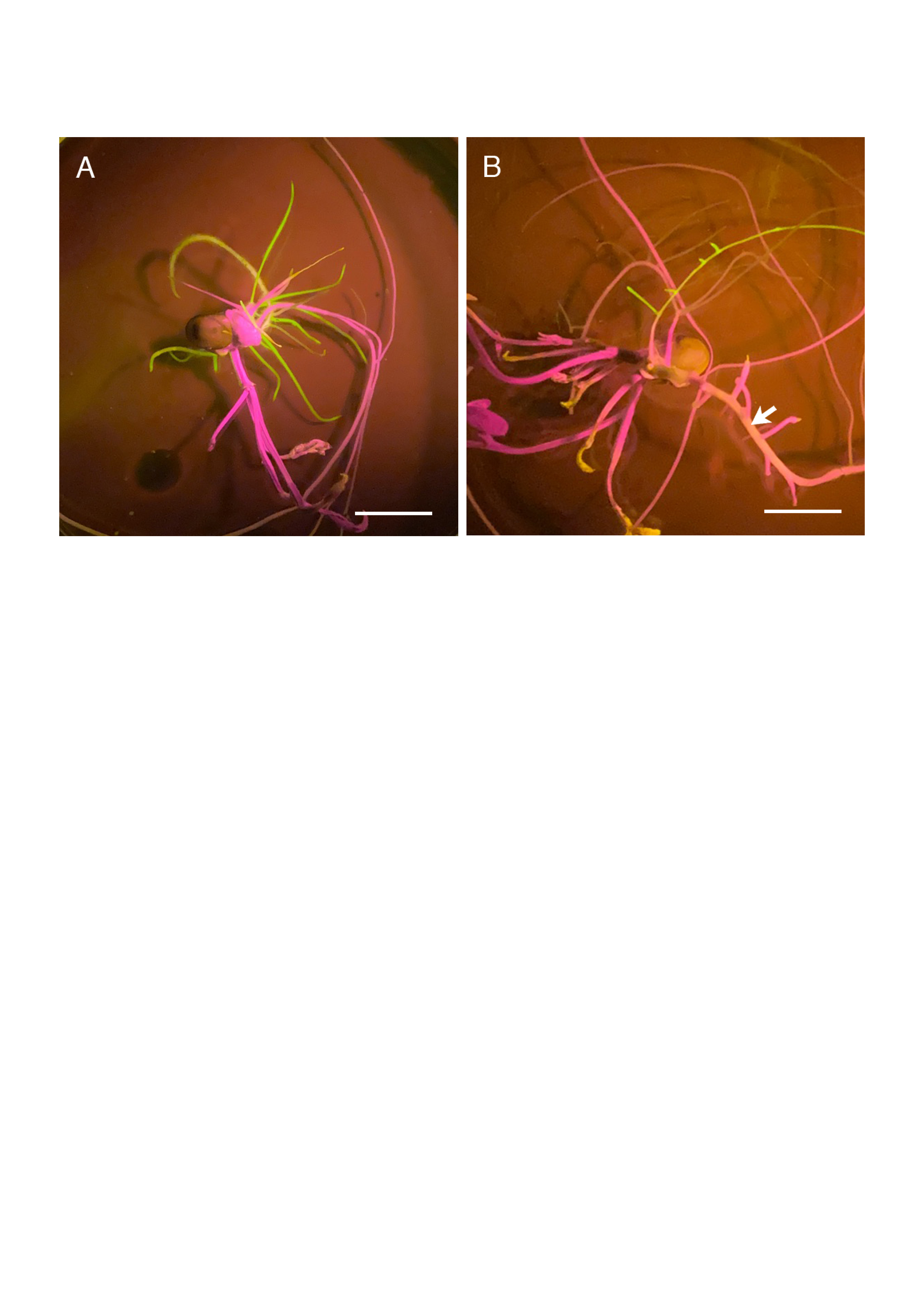

Supplement: Supplementary Figure 2 — GFP hairy root induction from (A) hypocotyl-epicotyl and (B) stabbed seedling. (A) Primary root was removed at day 0 of the infection with R. rhizogenes. (B) Primary root was kept during the hairy root induction period. GFP expressing transgenic hairy roots (green color) were detected using a hand-held blue light (Dark Reader Lamp – Clare Chemical Research), red color was the auto-florescence from tissue containing chlorophyll. White arrow indicates the wild type primary root. Scale bar = 1 cm. [file Image_2.JPEG]

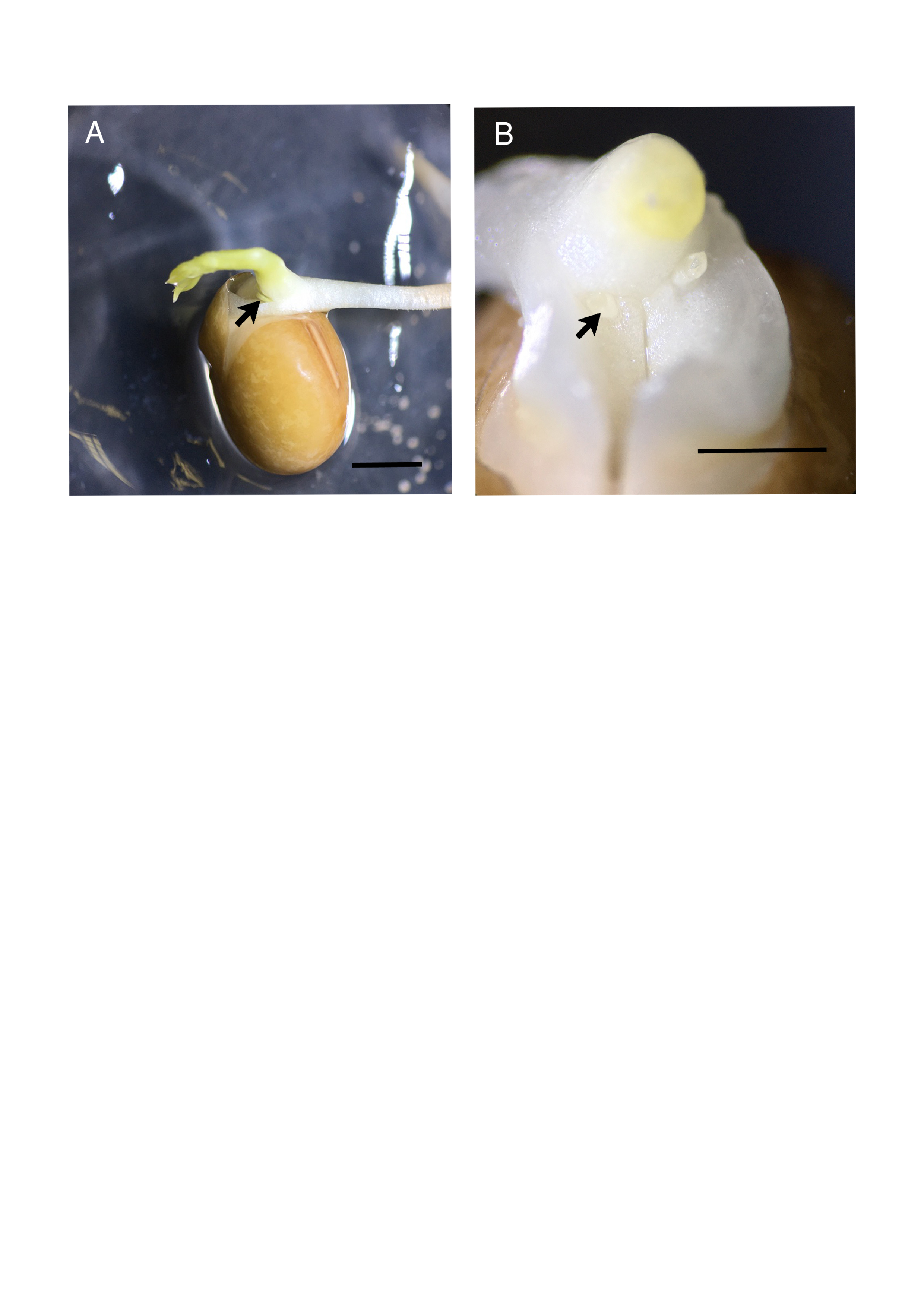

Supplement: Supplementary Figure 3 — Lateral shoots of vetch seedling. (A) Vetch seedling 5 days after germination in vitro on RGM_NoSuc media. (B) A close-up of a lateral shoot. Black arrows indicate the lateral shoot position at the junction of cotyledon and epicotyledon. Scale bar (A) = 1 mm; (B) = 0.5 mm. [file Image_3.JPEG]
