# Supplementary material for: An Efficient Root Transformation System for Recalcitrant Vicia sativa
Source: Front Plant Sci. 2022 Jan 7;12:781014. doi: 10.3389/fpls.2021.781014 (PMC8777216; doi:10.3389/fpls.2021.781014)
Supplement: Supplementary file 4 [file Table_1.docx]

**Supplementary Table 1**| Percentage of seedlings formed hairy roots after transfection with *R. rhizogenes* K599 using stabbing method under soil and *in vitro* condition. Hairy root induction efficiency was calculated 24 days after the infection. Each replicate was an independent transformation experiment in which data from 30 to 100 explants were collected. Percentages are mean ± SD for eight independent replicates.

| **Culture condition** | **Replicates** | **Percentage explant formed hairy root (%)** |
| --- | --- | --- |
| Soil | 8 | 49.48 ± 5.01 |
| *In vitro* | 8 | 18.81 ± 9.83 |
