# Supplementary material for: An Efficient Root Transformation System for Recalcitrant Vicia sativa
Source: Front Plant Sci. 2022 Jan 7;12:781014. doi: 10.3389/fpls.2021.781014 (PMC8777216; doi:10.3389/fpls.2021.781014)
Supplement: Supplementary file 5 [file Table_2.docx]

**Supplementary Table 2**| Percentage of explants formed hairy roots *in vitro* after transfection with *R. rhizogenes* K599. Hairy root induction efficiency was calculated 24 days after the infection. Each replicate was an independent transformation experiment in which data from12 to 54 explants were collected. Percentages are mean ± SD for three independent replicates.

| **Explant** | **Replicates** | **Percentage explant formed hairy root (%)** |
| --- | --- | --- |
| Seedling | 3 | 11.00 ± 6.22 |
| Hypocotyl-epicotyl | 3 | 92.66 ± 1.17 |
| Shoot | 3 | 100 ± 0.00 |
