# Supplementary material for: An Efficient Root Transformation System for Recalcitrant Vicia sativa
Source: Front Plant Sci. 2022 Jan 7;12:781014. doi: 10.3389/fpls.2021.781014 (PMC8777216; doi:10.3389/fpls.2021.781014)
Supplement: Supplementary file 6 [file Table_3.docx]

**Supplementary Table 3**| Average number of hairy roots per hypocotyl-epicotyl or shoot at 24 days after the transfection with *R. rhizogenes* K599. The number of hairy roots per explant was counted 24 days after the infection. The number of hairy roots per explant is mean ± SD for all the explants in the same group.

| **Explant** | **Number of explants** | **Number of hairy roots per explant** |
| --- | --- | --- |
| Hypocotyl-epicotyl | 21 | 4.19 ± 1.86 |
| Shoot | 12 | 4.25 ± 1.48 |
