# Supplementary material for: An Efficient Root Transformation System for Recalcitrant Vicia sativa
Source: Front Plant Sci. 2022 Jan 7;12:781014. doi: 10.3389/fpls.2021.781014 (PMC8777216; doi:10.3389/fpls.2021.781014)
Supplement: Supplementary file 7 [file Table_4.docx]

**Supplementary Table 4**| Co-transformation efficiency of *R. rhizogenes* K599 in hypocotyl-epicotyl and shoot explants. The co-transformation efficiency = percentage of hairy root with GUS activity in the total hairy root detected 24 days after the transfection. Percentages of GUS hairy roots/ explant are mean ± SD for all the explants in the same group.

| **Explant** | **Number of explants** | **Co-transformation efficiency (%)** |
| --- | --- | --- |
| Hypocotyl-epicotyl | 24 | 91.07 ± 16.21 |
| Shoot | 15 | 72.89 ± 19.82 |
